# Fire Escapes

**Published:** 1893-04-15

**Authors:** 


### PRACTICAL DEPARTMENTS.

### FIRE ESCAPES.

We are constantly being reminded by the sight of paragraphs in the daily papers headed "Fatal fires," of the necessity that exists for the provision of some method of escape from upper stories, in the event of the terrible calamity of a fire. It is to be hoped that, taught by sad experience in the past, most large institutions, hospitals, schools, &c., are now generally provided with escapes of some kind. Many are the cases where valuable lives might have been spared had there been even a rope at hand at the moment of peril. Various inventions for a speedy rescue have been brought out during recent years; one very generally in use in hotels and institutions is that known as the "Anidjah" (after the name of the maker). This is quickly put into action, and involves no risk in the descent. The apparatus consists simply of a long canvas shoot or tube, the upper opening of which completely covers the entire aperture of the window when in position for use, thereby preventing any person from falling out. The tube, by a chemical process, is made uninflammable, and if wished an absolutely fireproof sheath can be provided. The tube, which can be ordered of any length, is attached to an iron framework permanently fixed inside the window, and can be folded up and stowed away in a box ottoman. When needed, there is nothing more difficult to do than to pull it out of the box and throw the end of the tube out of the window. A rope runs down inside, enabling the first person to slide down quite easily without assistance from below; the descent would probably be more comfortably managed when the end can be held out in a slanting direction, but even without this really no one, however nervous, need fear to trust themselves on so quick and easy a journey. The price varies according to the length of shoot required; one twenty-four feet long costing £5. Particulars can be had from the offices of the "Anidjah Fire Escape Company," 254, High Holborn.

Basket fire escapes have their disadvantages, but one that has recently been brought under our notice, deserves some mention. The basket is made of wicker, strengthened with iron. A long rope is attached, firmly fixed to each side; when not in use this is coiled away and hidden by a false bottom, the basket being of such a size and shape that it can be used as a linen basket, and yet quickly converted into an efficient fire escape. For the descent, it is easily fixed by means of a pin and hook supplied with the apparatus, while the person gets into it, and the lowering is a matter of little difficulty. The last person to descend must pass the rope through the hook or round some piece of furniture, and drop the coil outside, then holding the rope, pay it out as he comes down. When needed for a very high window, it is advisable to have an iron frame fitted above the window, outside, to prevent the basket and its occupant from coming into contact with the wall. The inventor of this contrivance is a Berkshire clergyman, the Rev. Arthur Wilson. Any information can be had from A. James, Mortimer, Reading. As an article that 'Not only doth its end produce. But serves a second to some other use," this invention is one which is likely to be popular, especially as it has the additional advantage of being very reasonable in price; the whole arrangement complete can be obtained for 25s. It must be remembered, however, that there is the danger of the rope igniting, and in cases where in the event of a fire the escape would be needed for infirm or aged people, we should recommend the greater security of the one first mentioned. For private houses, the basket escape will, however, supply a real want.

